# Peripheral VA-ECMO and pericardial drainage connected to the ECMO circuit for cardiac tamponade from blowout rupture: a case report

**DOI:** 10.1186/s12872-023-03477-4

**Published:** 2023-08-31

**Authors:** Taichi Kato, Atsushi Miyagawa, Mayu Hikone, Koichi Yuri, Kazuhiro Sugiyama

**Affiliations:** 1 Tertiary Emergency Medical Center, Tokyo Metropolitan Bokutoh Hospital, 4-23-15 Kotobashi, Sumida-ku, Tokyo, 130-8575 Japan; 2 Department of Cardiovascular Surgery, Tokyo Metropolitan Bokutoh Hospital, 4-23-15 Kotobashi, Sumida-ku, Tokyo, 130-8575 Japan

**Keywords:** Blowout rupture, Cardiac tamponade, Extracorporeal membrane oxygenation, Extracorporeal cardiopulmonary resuscitation, Myocardial infarction

## Abstract

**Background:**

Left ventricular free wall rupture, particularly the blowout type, is still one of the most lethal complications of myocardial infarction and can cause catastrophic cardiac tamponade. Extracorporeal membrane oxygenation (ECMO) is often used to treat haemodynamic instability due to cardiac tamponade. However, elevated pericardial pressure can cause collapse of the right atrium, resulting in inadequate ECMO inflow and preventing the stabilisation of the circulation. Further, it can interfere with the venous return from the superior vena cava (SVC), increasing the intracranial pressure and reducing cerebral perfusion levels.

**Case presentation:**

A 65-year-old man was hospitalised for out-of-hospital cardiac arrest. We used ECMO for cardiopulmonary resuscitation. After the establishment of ECMO, transthoracic echocardiography and left ventriculography revealed massive pericardial effusion. The treatment was supplemented with pericardial drainage since ECMO flow was frequently hampered by suction events. However, the blowout rupture led to the requirement of constant drainage from the pericardial catheter. To tend to this leak, we connected the venous cannula of ECMO and the pericardial drainage catheter. The surgery was performed with stable circulation without suction failure of ECMO. During the course of the intensive care management, the neurological prognosis of the patient was revealed to be poor, and the patient was shifted to palliative care. Unfortunately, the patient died on day 10 of hospitalisation.

**Conclusion:**

We present a case wherein the combination of pericardial drainage and ECMO was used to maintain circulation in a patient with massive pericardial effusion due to cardiac rupture.

**Supplementary Information:**

The online version contains supplementary material available at 10.1186/s12872-023-03477-4.

## Background

Left ventricular free wall rupture is one of the most lethal complication of myocardial infarction that occurs in 1–4% of myocardial infarction case [1, 2]. Particularly, the blowout type rupture is usually fatal unless immediate surgical intervention is performed [3, 4]. Moreover, patients with blowout rupture usually present with severely unstable haemodynamics or cardiac arrest driven by cardiac tamponade, which requires immediate pre-surgery venoarterial extracorporeal membrane oxygenation (VA-ECMO). Increased pressure from the pericardial sac may prevent venous cannula drainage and ECMO flow [5–7]; hence, establishing VA-ECMO alone may be insufficient for pre-surgery circulatory management. Tamponade relief with pericardial drainage effectively reduces the pericardial pressure and eliminates excessive suction [8]. Herein, we report a case of blowout rupture that was complicated by cardiac tamponade, resulting in a cardiac arrest. The patient was managed with a novel combination of veno-venoarterial extracorporeal membrane oxygenation (VVA-ECMO) and pericardial drainage, pre-surgery.

### Case presentation

A 65-year-old man with chest pain for 5 days was transferred to our emergency room with out-of-hospital cardiac arrest. His medical history included hypertension and hypercholesterolaemia. A pulseless electrical activity was detected initially. After arrival, ultrasound examination revealed pericardial effusion. We attempted pericardiocentesis but failed to remove the excess fluid by pericardial drainage. We immediately opted for extracorporeal cardiopulmonary resuscitation (ECPR). We punctured the right femoral artery (16 Fr arterial cannula) and vein (22 Fr venous cannula) to establish VA-ECMO with a low flow time of 26 min.

**Fig. 1:**
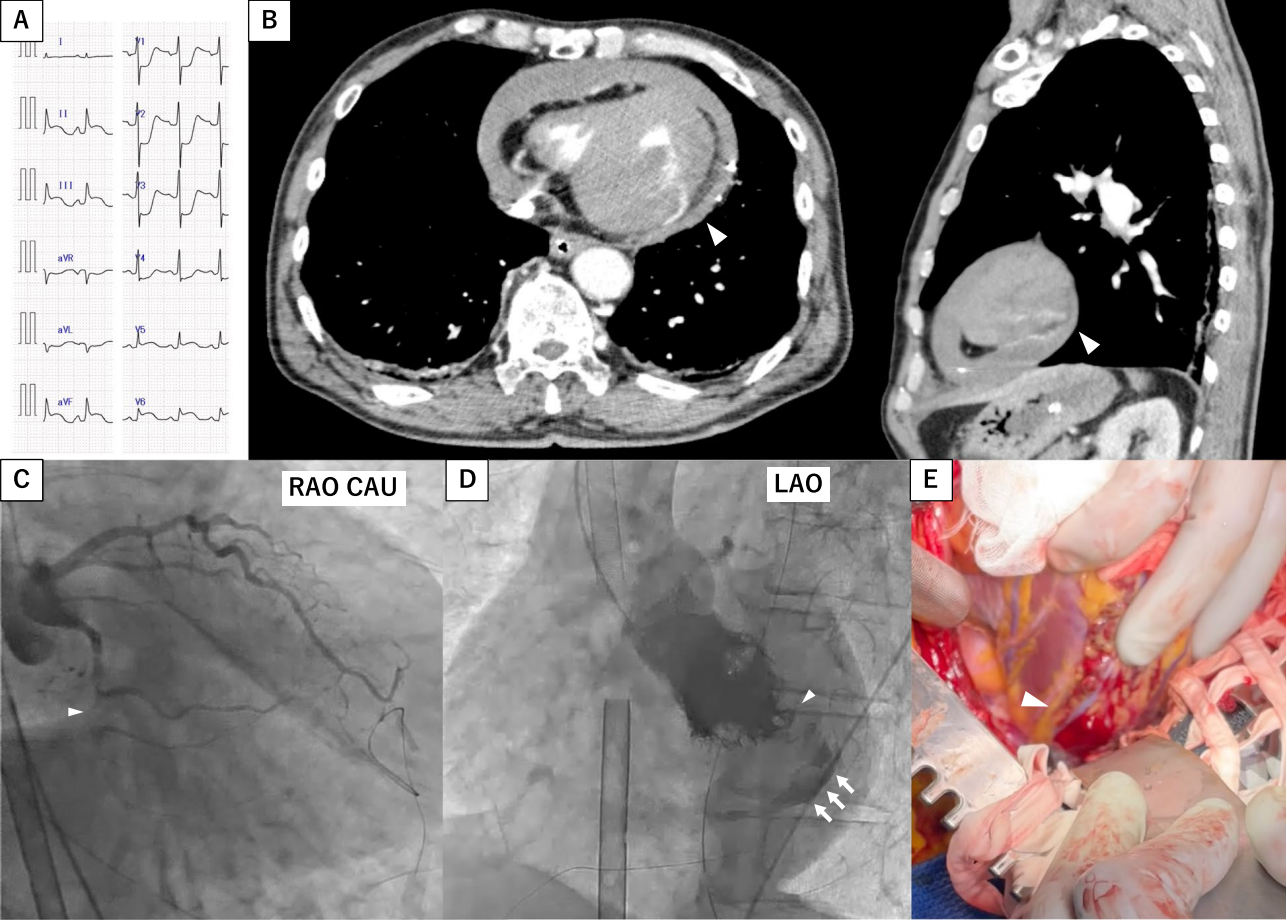
Initial examination findings. **a** Electrocardiogram at admission revealing ST elevation in leads II, III, aVF, and V4-6. **b** Enhanced computed tomography showing left ventricular rupture (arrowhead). **c** Coronary angiography showing 99% stenosis at LCx #13 (arrowhead). **d** Left ventriculography showing left ventricular rupture (arrowhead) and contrast residual dye within the pericardial space (arrow). **e** Intraoperative diagnosis of a transmural fissure at the inferoposterior wall. LCx, left circumflex branch

Electrocardiography revealed ST-segment elevation in leads II, III, and aVF (Fig. 1A). The placement of echo-guided pericardial fossa puncture and pericardial drain (8 Fr) (Argyle Fukuroi aspiration Seldinger kit; Cardinal Health, Dublin, United States) was performed. Further, 200 mL of bloody pericardial fluid was aspirated from the drain tube. Contrast-enhanced computed tomography (CT) revealed inferior left ventricular wall extravasation (Fig. 1B). Myocardial infarction-induced rupture was assessed using coronary angiography, which revealed 99% stenosis in the left circumflex branch #13 (Fig. 1C). A carefully manoeuvred left ventriculogram showed contrast leakage from the inferior left ventricular wall into the pericardial cavity (Fig. 1D, Video S1). Clinical and contrast findings indicated a blowout rupture diagnosis; therefore, open chest surgery was scheduled with total cardiopulmonary bypass equipment.

**Fig. 2:**
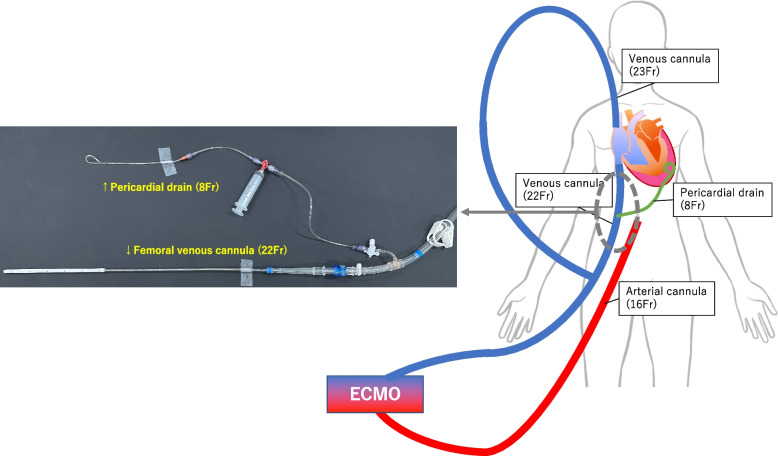
Combined mechanical support strategy for extracorporeal membrane oxygenation and pericardial drainage

**Fig. 3:**
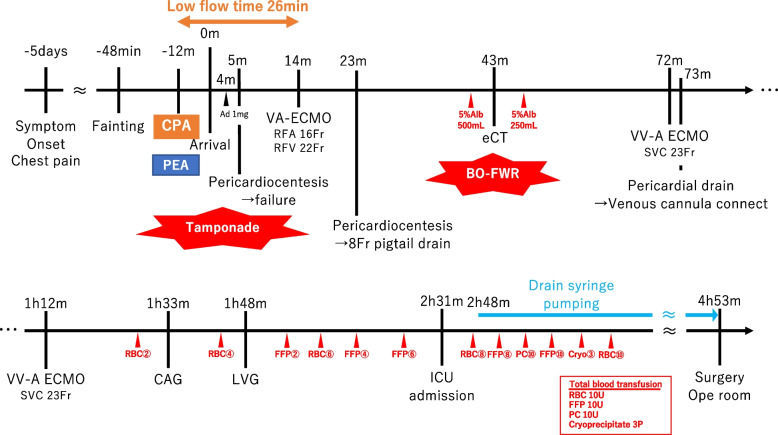
Timeline of rescue and management from symptom onset to surgery

After CT, the patient developed marked upper body congestion, which suggested impaired return from the superior vena cava (SVC). A venous cannula (23 Fr) was inserted into the right internal jugular vein and VA-ECMO was converted to VVA-ECMO. During surgical preparation, the ECMO venous pressure repeatedly dropped to as low as -150 mmHg; therefore, maintaining sufficient ECMO flow was challenging despite repetitive volume loading with multiple red blood cell, albumin, and fresh frozen plasma (FFP) transfusions. Echocardiography revealed continuous bleeding into the pericardial space. Reduction of the pericardial pressure and prevention of excessive suction were effectuated by directly connecting the pericardial drain to the ECMO venous cannula (Fig. 2). Continuous drainage allowed for pericardial sac decompression and prevented the high insufficiency of ECMO access present prior to the procedure, thereby reducing transfusion demand. Figure 3 summarises the procedure from the patient's arrival to the operating room. The patient received 0.16 gamma noradrenaline, 0.03 gamma adrenaline, and 3.3 gamma dopamine continuously.

Surgery was performed through median sternotomy. Femoral VA-ECMO was switched to total cardiopulmonary bypass for pericardial sac opening. Blood spurted out, while clots were removed when possible. Observation of the heart revealed a 1.5-cm penetrating mural fissure in the posterior wall that was surrounded by a broad necrotic myocardium (Fig. 1E, Video S2). Bovine pericardium was used to seal the rupture. Haemostasis was confirmed, a drain was placed, and the patient was returned to the intensive care unit (ICU). Transthoracic echocardiography after the patient returned to the ICU revealed a cardiac ejection fraction of about 20%.

A flat line was observed on the continuous electroencephalogram (EEG) after surgery, which transitioned to a burst suppression pattern 16 h after the cardiac arrest. On day 5 of hospitalisation, head CT showed indistinct corticomedullary cerebral cortex boundaries. The patient’s neurological prognosis was poor, and palliative treatment was suggested. On day 10 of hospitalisation, the patient underwent cardiac arrest while on ECMO, and death was confirmed.

## Discussion and conclusion

The prognosis of blowout left ventricular free wall rupture with cardiac tamponade or cardiac arrest is poor [9]. Since the condition of affected patients deteriorates rapidly, resuscitation for haemodynamic maintenance prior to surgical repair is crucial. Initial treatment options for free wall rupture pre-surgery generally include fluid loading, inotropic agent administration, mechanical support with intra-aortic balloon pumping or Impella (Abiomed, Inc., Danvers, MA, USA), and pericardial drainage [10]. During cardiac arrest, resuscitative measures generally include pericardial drainage. ECPR may be considered [10], especially when pericardial drainage and conventional cardiopulmonary resuscitation are ineffective.

Although ECMO is a feasible treatment for cardiac arrest, ECMO for cardiac tamponade has two potential problems. First, elevated pericardial pressure may lead to suction events and decreased ECMO flow in the event of cardiac tamponade [6]. Moreover, cardiac tamponade induced right atrial collapse, resulting in venous congestion at the inferior vena cava (IVC) or SVC, whichever was not canulated. Furthermore, increased intracranial pressure can occur and limit cerebral perfusion, especially when femoral VA-ECMO is established [11]. The patient who we report here could also have been resuscitated and operated upon in the acute phase; however, the neurological damage caused by hypoxic encephalopathy was fatal. The patient was eventually treated palliatively due to poor neurological prognosis.

In the current case, pericardial drainage successfully reduced the intrapericardial pressure and simultaneously maintained the ECMO flow. In a previous report, a pericardial drain connection with a central venous catheter was introduced for maintaining the VA-ECMO flow [8]. However, in our case, a pericardial drain was connected to the venous cannula of the VVA-ECMO. This facilitated rapid blood return via the pericardial drain, since the venous cannula of ECMO remained under negative pressure. However, a disadvantage of the technique is the increased probability of air embolisation during pericardial drain displacement. Another disadvantage is the aggravation of coagulopathy because of returning pericardial blood from outside of the vessel.

In addition to pericardial drainage, altering the positioning of the ECMO cannula can be effective. Here, VA-ECMO was initially established using a femoral venous cannula that was inserted into the IVC. Since significant upper body congestion occurred due to increased central venous pressure, a venous cannula from the SVC was added and VA-ECMO was converted to VV-A, which resolved the upper body congestion. Low flow time was 26 min; however exposure to high SVC pressure continued until SVC decompression at 84 min (Fig. 3). In this case, the neurological prognosis was poor, which influenced subsequent treatment decisions. It is unclear to what extent the high SVC pressure contributed to the poor neurological outcome by raising ICP and limiting cerebral perfusion. More rapid release of SVC congestion could, in theory, have contributed to a better neurological prognosis. One promising approach is raising the venous cannula tip to the SVC under fluoroscopy to establish VA-ECMO and ECPR. Alternatively, addition of a venous cannula from the SVC should be considered in the early phase.

In conclusion, resuscitation of patients with fatal collapsed blowout rupture followed by cardiac tamponade and arrest is often challenging. We have reported a case that highlights the importance of SVC suction and the advantages of connecting a pericardial drain to ECMO cannula for large pericardial effusions. In this case, preoperative resuscitative measures were performed using continuous pericardial drainage combined with VVA-ECMO for blowout rupture with cardiac tamponade. Initiation of VVA-ECMO with continuous pericardial drainage combined with an ECMO venous catheter may improve the haemodynamics prior to surgery.

### Supplementary Information

**Additional file 1: Video S1**. Left ventriculography showing a left ventricle rupture.

**Additional file 2: Video S2.** Ruptured scar of the posterior left ventricle wall during operation.

## Data Availability

All relevant data supporting the conclusions of this article are included within the article.

## Abbreviations

ECMO: Extracorporeal membrane oxygenation
ECPR: Extracorporeal cardiopulmonary resuscitation
EEG: Electroencephalogram
FFP: Fresh frozen plasma
ICU: Intensive care unit
IVC: Inferior vena cava
SVC: Superior vena cava
VA-ECMO: Veno-arterial extracorporeal membrane oxygenation
VVA-ECMO: Veno-venoarterial extracorporeal membrane oxygenation
